# Prevalence and risk factors of sexual harassment in the workplace by female readymade garment workers in Bangladesh

**DOI:** 10.3389/fpubh.2025.1580709

**Published:** 2025-05-22

**Authors:** Humayun Kabir, Myfanwy Maple, Kim Usher

**Affiliations:** ^1^School of Health, Faculty of Medicine and Health, University of New England, Armidale, NSW, Australia; ^2^Department of Sociology, University of Dhaka, Dhaka, Bangladesh

**Keywords:** sexual harassment, female workers, readymade garment sector, Bangladesh, quantitative

## Abstract

**Objectives:**

Bangladeshi female readymade garment (RMG) workers’ experience of sexual harassment in the workplace raises concerns about the overall working conditions of the garment sector. Female workers’ feelings of being unsafe and threatened in the workplace have been considered an alarming issue for international buyers/brands who aim to ensure sexual harassment-free workplaces as a condition of sourcing clothing items. We hypothesise that the frequent experience of sexual harassment among Bangladeshi female RMG workers tends to be associated with (i) age, (ii) marital status, (iii) night shift (working during night time), (iv) non-existence of anti-sexual harassment cells (a formal body/committee works against sexual harassment incidence in the workplace), (v) trade union activity, and (vi) factory location and types. To our knowledge, there has been no previous research on the experience of sexual harassment by female garment workers and its connections to such a variety of occupational and geographical factors. By addressing this gap, the present study aimed to investigate the prevalence and risk factors associated with female garment workers’ experience of sexual harassment.

**Methods:**

Cross-sectional data were collected from 332 (mean age = 26.10 years; SD = 6.54 years) currently employed female garment workers in Bangladesh, between February and July 2018. Data were analysed using bivariate and multivariate logistic regression modelling.

**Results:**

In the past 6 months, nearly one-quarter (22.0%) of workers reported experiencing frequent sexual harassment inside the factory, mainly by male co-workers (37.0%), supervisors (32.9%), security guards (27.4%), and factory owners (2.7%). Workers from the factories located in Chattogram (a peripheral region compared to Dhaka) reported a higher frequency of sexual harassment than those working in factories located in Dhaka (the capital city of Bangladesh). Overall, the percentages of unmarried and young female workers who experienced sexual harassment were almost double compared to married and aged female workers. Workers’ frequent experience of sexual harassment at the workplace was associated with factory location (*β* 0.67, 95%CI 1.02, 3.76), night shift (*β* 2.58, 95%CI 6.92, 25.18), and non-existence of an anti-sexual harassment cell inside the factory (*β* 0.62, 95%CI 0.97, 3.55).

**Conclusion:**

Urgent improvements in overall workplace conditions and anti-sexual harassment programmes are needed to safeguard female workers in the Bangladeshi RMG sector.

## 1 Introduction

The World Health Organisation (WHO) defines sexual harassment as any unwanted sexual behaviour or requests for sexual favour (1). It includes sexual jokes, unwelcome touching (e.g., pinching, patting, rubbing, and purposefully brushing up against another person), repeatedly asking for a date/sex, and making comments about body parts, appearance, and clothing (2). In addition, the International Labour Organisation (ILO) claims there are two more categories: ‘quid pro quo’ and ‘hostile working environment’. Quid pro quo sexual harassment is when a worker is threatened with loss of employment when they refuse to accede to a request for sexual favour, while hostile working environments include conduct in the workplace that is viewed as intimidating (3).

Violence against women, including sexual harassment, is a violation of women’s human rights (1, 4–6) and is a major public health challenge. Globally, female workers experience sexual harassment in the workplace, including in high-income/Western countries such as the United States (7, 8), Canada (9, 10), England (11), and Australia (12, 13), for example. According to ILO (3), over 33% of women worldwide and 37.7% of those in Southeast Asia report having experienced physical and/or sexual abuse at some point in their lives, and of the forms of abuse that women experience at work, sexual harassment and violence rank highest at 77%.

Regarding South and Southeast Asian countries, female workers were found to be more susceptible to workplace sexual harassment mainly because of the cultural and patriarchal structure of the society in those countries where males dominate females in every sphere of life, including the workplace and family life (5, 6, 14, 15). With a 25% female labour force participation rate, South Asia has the second-lowest rate of workplace harassment globally, where between 30 and 40% of women report having experienced sexual harassment at work (16).

Although female workers’ nimble hands, intensive, and cheap labour have played an active role in establishing the readymade garment (RMG) sector as the most important export-oriented and foreign exchange earning sector across the low- and middle-income countries (LMICS), workplace sexual harassment is particularly evident in the RMG factories (17). Therefore, the RMG sector located in the LMICS is criticism for failing to provide secure and safe working conditions for female workers. For example, in terms of country-specific data, 53.84, 22.9, and 42.5% of women employees/workers of garment workers in Nepal (18), Thailand (19), and Myanmar (20), respectively, reported experiencing sexual harassment in their workplaces. Orlando et al. (21) referenced a nationally representative survey in Bangladesh that reported one-third of women in the RMG sector reported sexual harassment in the workplace. The alarmingly high rate of sexual harassment against women employed in India’s garment industry is rated as 1 in 14 having suffered physical abuse and 1 in 7 having been sexually assaulted or coerced into performing a sexual act (22).

In Bangladesh, the RMG sector provides important opportunities to engage unskilled females with paid work; females constitute about 90% of the total labour force in this sector (23, 24). Female workers in the garment sector usually work long hours for low wages, often in unhealthy and unsafe working conditions due to the profit-maximising motivations of factory owners (25–27). Reports of sexual violence and sexual harassment in the RMG industry in Bangladesh are high (28). For example, Action Aid’s (29) research reveals that 80% of Bangladeshi RMG workers have either personally experienced or observed sexual abuse and harassment at work, and 90% of them believe their jobs are having a detrimental effect on their health. Consequently, female workers’ experience of sexual harassment is a growing concern for the global community that buys clothing items from Bangladeshi factories (25, 30).

### 1.1 Background and context of the study

Numerous studies have focused on sexual violence and harassment in the workplace in the Bangladesh RMG sector (5, 25, 31–34). These females are reliant on their employment to provide financial support for their families, while simultaneously being vulnerable to poor health and lack of safety in attending work (17). Research has repeatedly demonstrated that female RMG workers are vulnerable to various types of psychological issues (e.g., anxiety, sleeplessness, depression, self-blaming, trauma, and fear) due to experiencing different types of sexual harassment (including suggestive comments, demeaning remarks, unwelcome touching, grabbing and other physical assaults (such as raping)) at the workplace (31, 34, 35). Female workers experience sexual harassment in all areas of their lives including in their place of residence and on the way from their residence to the workplace, perpetrated by those who are known to them including husbands/partners, neighbours, landlords, security guards, work colleagues and those who are unknown to them including passersby, bus helpers/drivers, rickshaw pullers (15, 25, 34, 36). Intimate partner violence at home is also rarely reported due to the fear of being divorced by their husband if they do so, which leads to social stigma in Bangladesh (37). Similarly, reports of the experience of sexual harassment at the workplace are rarely made for fear of losing their jobs (25). In addition, factory owners tend to suppress sexual harassment issues that arise in the workplace so as not to tarnish their reputations (25). A study reported that female workers were threatened to lose their jobs if they exposed sexual harassment cases to the unions and media, and found they did not receive justice in cases where the incident was reported to the anti-sexual harassment cell that existed in their factories (25), because the majority members of the cell were males who protected other males in the factory.

Previous research conducted in Bangladesh has focused on female RMG workers’ experience of sexual violence in their homes and workplace (5), intimate partner violence (37), gender-based violence and harassment (38), violence and its relation to the development of depressive symptoms (15), and work–family conflict (39). These studies were conducted mainly in Dhaka-based (the capital city of Bangladesh) factories and/or nearby factories. However, a systematic review found that Chattogram-based (previously known as Chittagong, the second-largest city in terms of the number of garment factories) factory workers’ health vulnerability issues are further under-reported (17), demonstrating these issues extend beyond the capital city and into the regions where there is less international focus. In addition, a recent study identified correlations between RMG factory’s geographical location, factory type, workers’ age, marital status, active participation in trade union activity, night shift, and the existence of an anti-sexual harassment cell (a formal body/committee works against sexual harassment incidence) with RMG workers’ different types of vulnerabilities (35), which remained the central hypothesis of this study. Therefore, this study aims to address a significant limitation of previous research by investigating these factors in relation to the incidence of sexual harassment of female RMG workers. In addition, this study also explores the causes and consequences of experiencing sexual harassment in the RMG industry in Bangladesh.

### 1.2 The present study

To address the gap in the existing literature, we examined the prevalence of female workers’ experience of sexual harassment in the workplace setting. In addition, we examined a range of risk factors associated with experiencing sexual harassment in the workplace. The risk factors included workers’ demographic characteristics, geographical location of RMG factories, working nature, and trade union activity. In this study, we argue that female workers’ sexual harassment issues do not get proper attention and are kept unresolved year after year in the Bangladesh RMG sector due to the factory owners’ attitudes of ‘more profit through less investment’ which is compared with the process of ‘accumulation of capital’ as proposed by Marx (40). The concept ‘accumulation of capital’ was previously used by Kabeer and Mahmud (41) to specify the strategy of the factory owners to maximise their profits through the minimum possible cost. For this reason, RMG female workers become vulnerable as factory owners are unwilling to invest money in improving workplace conditions (i.e., workplace safety-related issues).

## 2 Methods

### 2.1 Study population and setting

The study was based on a cross-sectional survey among the currently employed female RMG workers recruited from the export processing zone (EPZ) and non-EPZ factories located in Dhaka and Chattogram cities of Bangladesh. The selection criteria of the target respondents include: (1) currently employed (during the time of interview) female workers, (2) aged 18 and above, and (3) with a minimum of 1 year of work experience in RMG factories. In addition, the exclusion criteria were as follows: (1) male workers, (2) factory management (manager, supervisors, and owners) and (3) pregnant women workers (to avoid expected risk during data collection and to comply with the ethics approval). Detailed methods and methodology on this study have been published elsewhere (35).

Using a purposive/convenience sampling method (justification of using this sampling method is listed at the end of this section), the study employed 332 (mean age = 26.10 years, SD = 6.54 years) females working in the sample, cutting, sewing, and finishing sections mainly. These working sections represent the common structure of the Bangladesh RMG sector (35, 42–44). Power analysis using Cohen’s (45) formula suggests a sample size of 200 provides an 80% chance of detecting a correlation ± 0.223 at 0.01 level. In addition, due to the sensitive nature of the study, the sample size remained lower. However, the sample size in our study remained higher compared to the previous studies conducted among Bangladeshi female RMG workers. For example, Sohani et al. (46) conducted a study among 180 Bangladeshi female workers to understand the pattern of sexual harassment. Uzzaman et al. (47) recruited 201 Bangladeshi female RMG workers to explore personal safety and fear of sexual harassment issues.

Written informed consent was collected from all of the respondents. Each respondent was gradually given a thorough, standardised explanation that included details on the study’s background and aim, the parties involved, survey processes, participant anonymity and confidentiality. Respondents gave their consent by signing the form and ticking yes/no on the relevant information contained in the ‘participant information’ sheet. Five respondents did not answer all of the 25 questions included in the survey questionnaire and were excluded from the study. Since 332 respondents gave their written consent, the response rate was 98.5%. The interviews were conducted between February and July of 2018 by the first author (who was fluent in Bangla/Bengali, the mother language of both the interviewer and interviewees) and lasted between 60 and 75 mins.

The labour union offices close to the clothing factories served as the interview locations. The first author had prior work experience in the Bangladesh RMG sector, and thus, he contacted RMG trade union leaders and currently employed workers to assist with the recruitment of RMG workers. The trade union leaders, the pre-acquainted workers, and the first author himself then made phone calls to prospective respondents for interview purposes. Thus, the total number of factories was not the prime concern, as the interviews were not conducted inside the factory premises. This provided the authenticity of the research to potential participants and also ensured safety for those who decided to take part in the research. None was allowed to enter during the time of the interview to maintain privacy. Recruitment was finalised once the required number of female respondents had been interviewed. Finally, the respondents were interviewed from 121 factories, where at least one respondent from each participating factory was confirmed as a participant.

### 2.2 Measures

#### 2.2.1 Risk factors

Risk factors such as factory location and factory type were categorised as ‘Dhaka and Chattogram’, and ‘EPZ and non-EPZ’, respectively, to cover the whole RMG sector of Bangladesh. The marital status of the respondents was categorised as ‘unmarried’ and ‘married’. The education level of the respondents included the following categories: ‘up to primary’ and ‘secondary and above’. Response categories for age were kept open in the survey instrument and were later categorised into ‘up to 25/above 25’. In addition, night shift (performing duties during night time), the existence of the anti-sexual harassment cell inside the factory, and female workers’ active participation in trade union activity were measured by ‘no/yes’ alternatives. The location and type of the RMG factories, marital status, education level, age, night shift, existence of the anti-sexual harassment cell inside the factory, and female workers’ active participation in trade union activity were previously identified as risk factors in studies conducted by Uzzaman et al. (47) and Kabir et al. (35); Fitch et al. (48); Lu and Lu (49); and Kabir et al. (25), respectively.

#### 2.2.2 Outcome variable

The outcome variable (experience of sexual harassment) was measured by asking a general question, ‘How often do you experience sexual harassment inside the factory in the last six months?’ using a dichotomous response option, such as frequently/not frequently. ‘Frequently’ was defined as the regular happening and/or everyday experience, and ‘not frequently’ was identified as not an everyday experience in line with the pilot study recommendations. In addition, the participants of this study were given a clear idea about what was meant by ‘frequent sexual harassment’ - as any unwanted sexual behaviour that occurs at least once per day. The self-rated answer and 6-month timeframe were previously used in similar types of research conducted in the Bangladesh RMG sector (35, 42).

### 2.3 Procedure

As mentioned earlier, the survey questionnaire was developed from the existing literature and validated by the experts (the authors) in the fields of sociology, social work, and mental health. The authors previously conducted a systematic literature search to understand the overall scenario of the RMG sector located in the South and Southeast Asian countries, including Bangladesh (17). The ideas of the current research were developed from the insights of a previously conducted systematic review. In addition, in January 2018, the interview items along with the response categories were pilot-tested with 15 workers recruited from both Chattogram and Dhaka cities, who were not included in the final sample utilised for the analyses. The recommendations from the pilot study were taken into consideration for further refining the survey questions. The knowledge and expertise gained from the systematic review and pilot study enabled the two co-authors to validate the survey questionnaire. The first author translated the English survey questionnaire into Bangla/Bengali. A bilingual expert from the University of Dhaka in Bangladesh approved both Bangla and English versions of the survey.

### 2.4 Statistical analyses

All data from paper copies were entered into SPSS (version 25) by the first author. Initially, chi-squared tests were used to investigate the associations between a number of risk factors and the outcome variable (frequent experience of sexual harassment). Then, in order to determine whether risk factors were substantially linked to the occurrence of sexual harassment among female workers, logistic regression analyses were performed. We employed those predictor variables as risk factors in the multivariate analyses that demonstrated significant bivariate relationships (*p* < 0.05) with the outcome variable. The important determinants of the experience of sexual harassment indices are interpreted using adjusted odds ratios (ORs) with 95% confidence intervals (CIs). In addition, a variety of model fit indices (including the omnibus test of the model, Nagelkerke *R*^2^, Hosmer and Lemeshow *R*^2^, and Cox & Snell *R*^2^) were used for the outcome variable.

### 2.5 Justification of using a purposive/convenience sampling method

There is no doubt that systematic or random sampling is methodologically more robust and acceptable than convenience sampling. However, this sampling procedure was not applicable to our study, given the fact that the whole population is unknown and the recruitment of the respondents was not possible without assistance from the factory management. We intentionally avoided using random sampling procedures because the researchers had to get permission/assistance from the factory management to reach workers if random sampling was followed, be that for workers’ names, list/ID, and other required information. In that scenario, the respondents might feel pressured to take part in the survey or to provide specific (biased) responses. As a result, there was a chance that this technique would have been biased—the presence of factory management during the interview would have affected the respondents’ answers in a true meaning. This is why interviews were conducted outside of factories to obtain accurate and reliable responses from the respondents.

To ascertain whether the survey questionnaire was acceptable, a pilot study was conducted. This was undertaken in one factory. The respondents were reluctant to talk about issues related to their health, wages, and working conditions. The same respondents provided different answers (realities) about the same topic of interest when they were interviewed by the same person outside the factory. Furthermore, the first author had to use the snowball technique due to increased monitoring, surveillance, and barriers (posted by factory owners) that prevented journalists and researchers from accessing currently employed workers in the workplace, particularly following the collapse of the Rana Plaza in 2013 (23, 42, 48, 50). In addition, for reasons of safety and security, the University of New England Human Research Ethics Committee refused to allow the first author to visit the residences of RMG workers. Moreover, there were no specific areas where only RMG workers resided. In Bangladesh, there are semi-slum/slum areas where the majority of residents, who are RMG workers, reside. Convenience snowball sampling was deemed appropriate for the current study in light of these considerations.

## 3 Results

### 3.1 Sample characteristics

The study sample characteristics are presented in Table 1. The mean age, working years, monthly income in BDT (Bangladeshi Taka) without overtime, and monthly income with overtime were 26.10 years (SD = 6.54), 5.84 years (SD = 4.79), BDT 7909.73 (SD = 2157.32), and BDT 10302.11 (SD = 2560.46), respectively. The overwhelming majority of the respondents originated from a rural area (93.4%), had low educational backgrounds (58.7% had up to class 5, which is known as primary school education level), and most were married (77.4%). The respondents were recruited equally based on the location and type of factories. The main garment process was sewing (60.2%).

**Table 1:** Summary of sample characteristics (*N* = 332).

Demographic variables		Value
Age in years, mean ± SD (range)	26.10 ± 6.54 (18–55)
Working years in the RMG sector in years, mean ± SD (range)	5.84 ± 4.79 (1–29)
Monthly salary in BDT (without overtime), mean ± SD (range)	7909.73 ± 2157.32 (4000–17,500)
Monthly salary in BDT (with overtime), mean ± SD (range)	10302.11 ± 2560.46 (4000–21,000)
	*n* (%)
Geographic location	
Rural	310 (93.4%)
Urban	22 (6.6%)
Education	
Up to Primary (class 1–5)	195 (58.7%)
Secondary and above (above class 6)	137 (41.3%)
Marital status	
Unmarried	75 (22.6%)
Married	257 (77.4%)
Factory location	
Chattogram (previously known as Chittagong)	166 (50.0%)
Dhaka	166 (50.0%)
Factory type	
Non-EPZ	166 (50.0%)
EPZ	166 (50.0%)
Working section	
Sample	12 (3.6%)
Cutting	18 (5.4%)
Sewing	200 (60.2%)
Finishing	38 (11.14%)
Other (Helper, Dyeing, Ironing, Printing, Washing plant, Quality controller)	64 (19.3%)

*n* = sample size; RMG, readymade garment; BDT, Bangladeshi taka/currency; EPZ, export processing zone.

Table 2 shows that nearly one-quarter (22.0%) of the respondents experienced sexual harassment frequently during the last 6 months, mainly by male co-workers (37.0%), supervisors (32.9%), security guards (27.4%), and factory owners (2.7%). In addition, one-third (31.9%) of the respondents reported that there was no existence of anti-sexual harassment cells in their factories. The overwhelming majority of the respondents (81.6%) reported being unwilling to join the trade union mainly because of the associated social stigma (21.1%), fear of loss of job/job restriction imposed by factory owners (20.8%), not being aware of the existence/usefulness of a trade union (18.7%), or because they did not feel the necessity to join in trade union activities (16.0%).

**Table 2:** Experience of frequent sexual harassment and associated risk factors.

Sexual harassment and associated risk factors	*n* (%)
Frequent experience of sexual harassment inside the factory:	73 (22.0%)
Experience of sexual harassment inside the factory by	
Male co-workers	27 (37.0%)
Supervisor/Floor in charge	24 (32.9%)
Security guard	20 (27.4%)
Factory owner	2 (2.7%)
Absence of anti-sexual harassment cell in the factory	106 (31.9%)
Non-involvement in trade activity	271 (81.6%)
Reasons for non-involvement in trade union activity	
Social stigma of joining in trade union activity	70 (21.1%)
Fear to lose job/job restriction	69 (20.8%)
No idea about union	62 (18.7%)
Do not feel the necessity	53 (16.0%)
No time	17 (5.1%)
Night shift	
Yes	93 (28.0%)

*n* = sample size.

In the following section, we present the results of risk factors associated with female workers’ frequent experience of sexual harassment inside the factory premises. We conducted bivariate analyses followed by multivariate statistical tests.

### 3.2 Risk factors associated with frequent experience of sexual harassment

We conducted chi-squared analyses to examine the relationships between a range of risk factors and the experience of sexual harassment. Table 3 shows that unmarried and young female workers experienced more frequent sexual harassment compared to married and older female workers. Results found that factory location was significantly associated with the incidence of sexual harassment (see Table 3). Overall, respondents from the factories located at Chattogram reported more frequent experiences of sexual harassment (Chattogram vs. Dhaka: 27.1% vs. 16.9%) compared to the respondents from Dhaka. In addition, workers who had to work during a night shift experienced more sexual harassment compared to those who did not work during a night shift (night shift vs. no night shift: 58.1% vs. 7.9%). The frequency of experiencing sexual harassment increased among the factory workers where there was no anti-sexual harassment cell (Existence of anti-sexual harassment cell vs. no Existence of anti-sexual harassment cell: 14.9% vs. 33.1%). Similarly, workers who were not active in trade union activities reported a more frequent experience of sexual harassment compared to those who had active participation in trade union activities (active participation in trade union activity vs. non-active trade union activity: 16.4% vs. 23.2%).

**Table 3:** Summary of chi-squared analyses: relationships between risk factors and experience of frequent sexual harassment inside the RMG factory.

Predictors	Frequent experience of sexual harassment inside the RMG factory, n (%)
Age	***p* = 0.001**
Up to 25 years	54 (28.3%)
26 and above	19 (13.5%)
Education	***p* = 0.613**
Up to primary	41 (21.0%)
Secondary and above	32 (23.4%)
Marital status	***p* < 0.001**
Unmarried	28 (37.3%)
Married	45 (17.5%)
Factory location	***p* = 0.024**
Chattogram	45 (27.1%)
Dhaka	28 (16.9%)
Factory type	***p* = 0.145**
Non-EPZ	31 (18.7%)
EPZ	42 (25.3%)
Night shift	***p* < 0.001**
Yes	54 (58.1%)
No	19 (7.9%)
Existence of anti-sexual harassment cell inside the factory	***p* < 0.001**
No	43 (33.1%)
Yes	30 (14.9%)
Active participation in trade union activity	***p* = 0.243**
No	63 (23.2%)
Yes	10 (16.4%)

*n* = sample size; EPZ, export processing zone [Different risk factors were compared among the respondents who had experienced frequent sexual harassment (*n* = 73)].

Table 4 represents logistic regression analyses conducted to examine which specific risk factors were significantly associated with sexual harassment outcomes. Specifically, female workers who were employed at the garment factories located at Chattogram (factory location, *β* 0.67 95%CI 1.02, 3.76) and worked during the night time (night shift, β 2.58 95%CI 6.92, 25.18) were found to be the most likely to experience sexual harassment. Lastly, female workers whose factory lacked the existence of an anti-sexual harassment cell (β 0.62 95%CI 0.97, 3.55) reported significantly more experiences of sexual harassment (Table 4).

**Table 4:** Summary of logistic regression analyses: risk factors associated with frequent experience of sexual harassment inside the RMG factory.

Predictors	Frequent experience of sexual harassment inside the RMG factory *B* Odds Ratio, 95% CI
Age	
Up to 25 years	0.56 1.76 [0.82, 3.76]
Above 25 years	1
Marital status	
Unmarried	0.42 1.52 [0.70, 3.33]
Married	1
Factory location	
Chattogram	0.67 1.96 [1.02, 3.76]^*^
Dhaka	1
Night Shift	
Yes	2.58 13.21 [6.92, 25.18]^***^
No	1
Existence of anti-sexual harassment cell in the factory	
No	0.62 1.85 [0.97, 3.55]^*^
Yes	1
Cox & Snell *R*^2^	0.27
Nagelkerke *R*^2^	0.42
Hosmer and Lemeshow *R*^2^	0.75
Omnibus Model test^+^	χ^2^(5) = 104.84^***^

B, standardised beta coefficient; CI, confidence interval; RMG, readymade garment; ^*^p < 0.05; ^**^p < 0 0.01; ^***^p < 0.001.

## 4 Discussion

The purpose of this study was to determine the extent to which female RMG workers experienced sexual harassment at work and the relative contributions of the nature of their work, factory type, and factory location. To the best of our knowledge, this is one of the few studies addressing the frequency, risk factors, and experiences of sexual harassment across various types of factories (EPZ vs. non-EPZ) situated in various Bangladeshi locations (Dhaka vs. Chattogram). In summary, our research adds two distinct perspectives to the current body of relevant literature. First, this study compared the frequency of sexual harassment experienced by EPZ and non-EPZ workers; this difference had never been previously reported. Second, the study examined the differences in the incidence of sexual harassment experienced by RMG workers in Dhaka (the centre) and Chattogram (the peripheral relative to Dhaka)—a first attempt in Bangladeshi RMG-related research (17).

### 4.1 Prevalence of sexual harassment

Specifically, our study revealed that 22% of the respondents experienced frequent sexual harassment inside the factory premises, and 32.9% of them reported this harassment by their supervisors. A previous study conducted by Uzzaman et al. (47) reported similar findings, where it was found that 13.4% (very often) and 18.4% (often) of the female workers reported experiencing sexual harassment in the workplace. However, another study conducted by Sohani et al. (46) reported that 68.9% of female workers experienced psychological harassment, of whom 7.2% reported experiencing sexual harassment.

### 4.2 Risk factors associated with the experience of sexual harassment

The present study found that factory location was an important risk factor for experiencing sexual harassment by female RMG workers. RMG workers from the Chattogram-based factories experienced sexual harassment more frequently than the workers from the Dhaka-based factories. Similarly, previous studies found that garment workers located in the centre (such as Dhaka) had better working conditions (25) and wages (26), and were less vulnerable to physical and psychological health problems (17, 35) compared to the workers in areas away from the major cities. This is mainly because the representatives of international buyers/brands, ILO officials, and members of INGOS rarely visit the peripheral factories to avoid travel challenges and safety issues associated with travelling to factories out of the main cities (25, 26). The findings from this study highlight the need for greater oversight of factories outside major city areas as a way to improve working conditions for female RMG workers.

In consideration of export processing zones, those from the EPZ factory workers were found to experience more frequent sexual harassment compared to non-EPZ workers. This finding was surprising given that all EPZ factories report better working conditions, wages, foreign ownership and stricter monitoring practices (41). Since EPZ factories provide better salaries and more work-related facilities (such as medical care, available hygiene items and safety equipment, regular break between works and day off, emergency exists, fire alarms, and fire extinguishers), this could be the reason female workers do not wish to lose their jobs by reporting sexual harassment to the authorities. It is not unusual for female workers to lose their jobs due to reporting sexual harassment incidents at the workplace (25). In addition, a study showed that in many workplace settings, the justice system is often set up to fail, with victims required to report claims of experiencing sexual harassment through a strict working environment that might include the perpetrator or their allies (51). We argue that this might lead the perpetrator harass female workers of the EPZ factories. Repeated denial – thinking that sexual harassment does not happen in, say, strictly monitored RMG factories (such as EPZ) or that the EPZ authorities have already solved it – could be a false narrative that provides fertile ground for sexual harassment to thrive. This issue should be investigated in future studies and may be related to the pressure of the export businesses that are not present in the non-EPZ factories. However, similar to our study, another study found that EPZ workers were more vulnerable to depression, fear, and sleeplessness compared to non-EPZ workers (35).

Our study also revealed that the experience of sexual harassment varied in relation to the marital status of the respondents. In general, unmarried and young female workers were significantly more vulnerable to frequent sexual harassment compared to married and older female workers, which is consistent with the previous studies conducted in the Bangladesh RMG sector (5, 29, 52, 53). Previous research identified Bangladeshi young/unmarried female RMG workers as docile (easy to control) and immature compared to older and married female workers (33), which is why they might be easily targeted by the harassers. As a result, the issue of harassment of young, unmarried female workers must be addressed as a priority.

The length of service in RMG factories is naturally short due to the damaging working conditions to individuals’ health and wellbeing (35). Protecting young females who enter employment in these factories is a priority for the businesses to remain viable, and more importantly, for the well-being of young women.

The absence of anti-sexual harassment cells in the factories and female workers performing night shifts was identified as one of the most significant risk factors associated with female workers’ experience of sexual harassment in the workplace. While Kabir et al. (25) found that the presence of an anti-sexual harassment cell did not equate to safety in the workplace, and the existence of these has failed to prevent sexual violence and harassment in the workplace (54), nevertheless, where such cells are absent, workers are even more vulnerable. Kabir et al. (25) also noted that female workers suffered from anxiety and feeling unsafe while working during night shifts. Similarly, a study conducted by Lu and Lu (49) provides evidence that night shift duty was associated with the occurrence of mental health symptoms among Filipino female factory workers. In addition, another study identified how the experience of sexual harassment among female RMG workers led to depression (15). Anxiety and depression might lead female workers to develop suicidal thoughts and behaviour (55–57). Studies also found that heavy mental distress (which might be associated with sexual harassment and the lack of opportunity to report the incident) may cause poor sleep or sleeping disorders among garment workers (35, 58).

There are many challenges to female workers’ safety, and such concerns for personal safety and fear of sexual harassment may discourage women from participating at work and in public life, limiting their lifetime opportunities (47). Yet, the RMG sector provides opportunities that previously did not exist for unskilled young females to access economic security (albeit below a living wage). The factory owners must ensure the workers’ safety to continue working in such a significant industry in Bangladesh. Without this, the Bangladesh RMG sector risks losing its current ranking (currently 2nd, only after China) in the global supply chain and/or losing the opportunity to supply clothing items for the world, which is the main foreign exchange earning sector for Bangladesh.

In line with the study findings, we argue that female workers’ experience of sexual harassment is linked to the existing poor working conditions in the Bangladesh garment industry. Unfortunately, the existing poor working conditions benefit the garment owners. Marx’s (40) concept ‘accumulation of capital’ explains the relationship between the existence of poor working conditions and the experience of sexual harassment. For example, ‘the lesser the investment, the higher the profit’ is a basic economic equation. It is important to begin with how RMG factory owners achieve this outcome. They do so primarily by limiting costs associated with the needs of their workforce to keep them safe and secured by limiting workplace compliant issues (such as uses of CCTV in workplace, forming anti-sexual harassment committee with internal and external members, separate bus services for bringing and returning female workers from their living places, avoiding night shifts, separate toilet facilities for female workers) at the workplace. More specifically, we argue that RMG factory owners’ ‘less investment’ tendency is the main cause of the Bangladesh RMG sector’s existing poor working conditions, which eventually makes female workers vulnerable to experiencing sexual harassment. Given the vast unemployed female population of Bangladesh and because of the lack of employment options and poverty, factory owners can continue to act with less investment tendency, as when a female worker quits work, there is another waiting to take her working position.

### 4.3 Limitations and strengths

We have focused on the female RMG workers’ frequent experience of sexual harassment in workplace settings. We did not focus on the nature/types or the discrete number of sexual harassment incidents faced by female RMG workers. In addition, these workers may be at risk of facing sexual harassment in other settings, such as their homes, and on their way to the workplace, which was not a priority in our study. Respondents reporting experience of frequent sexual harassment were asked about who (e.g., male co-workers, supervisor/floor in charge, security guard, and factory owner) perpetrated the sexual harassment. Respondents were able to indicate only one perpetrator who was mostly relevant to their sexual harassment experience. By doing this, we ignored the very possibility that women may experience sexual harassment from more than one perpetrator in the factories. The use of purposive sampling may lead to selection bias. The survey questions used in this study lacked validation from other relevant experts in the field. Finally, the present study did not use any Western-validated measures to assess psychological health, as these were inappropriate for the population under investigation. Therefore, the results of the present study need to be interpreted with caution.

Notwithstanding these limitations, the reliability and validity of the data were maximised by drawing the items for the survey from previously reported literature, screening the questionnaire by four experts, attesting the survey instrument (both Bangla and English) by independent persons, conducting a pilot study, and collecting responses from the respondents through rigorous rapport building procedures (and verbally to allow for low literacy). Additionally, the large sample size, inclusion of female workers from EPZ and non-EPZ factories located at Dhaka and Chattogram cities, and response rate make the findings of the study relevant across the garment industry of South and Southeast Asian countries. Lastly, the inclusion of the female RMG workers who were recruited from 121 factories from all over Bangladesh is a strength of this study, and the findings should be considered rigorous by the relevant stakeholders.

### 4.4 Future research directions

Since sexual harassment and violence issues of Bangladeshi female RMG workers remain a great concern (5, 15, 25), further research should be initiated to explore the depth of this issue using more in-depth/narrative types of methodology to unveil the reality behind this issue. More specifically, risk factors associated with female RMG workers’ experience of sexual harassment inside and outside the factory premises could also be explored.

Future research may also seek to explore the impacts of sexual harassment on female RMG workers in Bangladesh and other relevant countries. Future studies may aim to compare and contrast the experience of sexual harassment of female RMG workers between developing and developed country perspectives and/or among developing countries. The role of factory management, international brands/buyers, INGO’s, ILO’s, and the relevant government agencies in establishing sexual harassment-free workplaces should also be explored. Future studies may include different stakeholders (e.g., representatives from the international brands/buyers, factory management, government of Bangladesh, NGOs, INGOs, and trade union leaders) along with the currently employed RMG workers.

## 5 Conclusion

Our study revealed that female RMG workers’ experience of sexual harassment in the workplace needs to be addressed. All the risk factors associated with sexual harassment indicates that the existing working conditions are unsafe for female RMG workers. If urgent actions are not taken to address female workers’ sexual harassment issues, this sector may lose its majority workforce, and the currently employed female workers may be susceptible to depression, anxiety and suicidal behaviour. Numerous studies conducted in a range of industries found that the experience of sexual harassment in the workplace is associated with a reduction in women’s job satisfaction (see (59–61)). Work disengagement is an important and frequent result of sexual harassment, and it can happen in several ways. Female workers who experience sexual harassment frequently take a leave of absence from their jobs, remaining with their employers but not participating in their work (62). Many employers often tend to protect and safeguard the unacceptable behaviour (such as sexual harassment) of the high performers at all costs, thinking that the company’s overall benefits (profits) are dependent on them. A gender-harassing power dynamic is perpetuated by narratives about the ideal men worker and the ideal homemaker-breadwinner. These narratives are based on the idea that males should not be providing care and that women and other nonconforming groups do not belong in the workforce. In addition to fuelling the frequency of sexual harassment, these harmful myths also create toxic and abusive cultures that mute, ignore, and waste the abilities and potential of innumerable female workers (51).

There were no legally binding international agreements for nations to enact national legislation and successfully combat workplace violence (38). In June 2019, the ILO adopted the Violence and Harassment Convention, 2019 (C190) to help its member states recognise and outlaw workplace harassment and violence (63). C190 urges all ILO members to enact relevant laws and rules so that employers are required to act to safeguard employees from harassment and violence (Article 9). Article 4 of this agreement mandates that member states consult with employers and workers’ organisations before implementing an inclusive, integrated, and gender-responsive approach for the prevention and elimination of violence and harassment. It is time to impose the ILO convention strictly in countries where female RMG workers experience frequent sexual harassment.

Finally, interventions must aim to reduce the frequency of sexual harassment in the workplace to zero level acceptance. Albeit, female workers’ human rights (sexual violence-free workplace) must be safeguarded in collaboration with RMG female workers, factory management, state, and relevant stakeholders by engaging more female workers in leadership positions (such as managers) and positioning them in the anti-sexual harassment cell for sexual harassment prevention.

## Data Availability

The raw data supporting the conclusions of this article will be made available by the authors, without undue reservation.
